# Genome sequences of bacteriophages that infect *Salmonella* Typhi from Bangladesh

**DOI:** 10.1128/mra.00447-24

**Published:** 2024-12-17

**Authors:** Shuborno Islam, Rathindranath Kabiraj, Himadree Sarkar, Preonath Chondrow Dev, Afroza Akter Tanni, Deb Purna Keya, Apurba Rajib Malakar, Arif Mohammad Tanmoy, Samir K. Saha, Yogesh Hooda, Senjuti Saha

**Affiliations:** 1 Child Health Research Foundation, Dhaka, Bangladesh; 2 Department of Microbiology, Bangladesh Shishu (Children) Hospital and Institute, Dhaka, Bangladesh; DOE Joint Genome Institute, Berkeley, California, USA

**Keywords:** *Salmonella*, bacteriophages, typhoid, genomics

## Abstract

This report presents near-complete genome sequences of 14 bacteriophages that infect *Salmonella* Typhi, identified through environmental surveillance in Bangladesh between August 2021 and June 2022. The bacteriophages, belonging to the genera *Kayfunavirus*, *Macdonaldcampvirus*, and *Teseptimavirus*, exhibit high degrees of sequence similarity and conserved genetic features with previously reported Typhi bacteriophages.

## ANNOUNCEMENT

*Salmonella enterica* serovar Typhi causes typhoid fever and remains a major public health concern in low- and middle-income countries (1). Historically, *Salmonella* Typhi-specific bacteriophages were used to characterize strains of *Salmonella* Typhi (2–4). Despite a decline in their use post-1980s, recent findings from Nepal (5) and Bangladesh (6) confirmed the persistence and genomic diversity of Typhi phages in endemic settings. This study reports sequences of 14 Typhi phages isolated in Bangladesh, expanding the global database.

Here, bacteriophages were isolated from surface water samples (Table 1) using *Salmonella* Typhi BRD948 strain grown at 37°C as the host, as described previously (6). Briefly, phages were amplified from single plaques, and pure phage lysates were treated with DNase, RNase, and Proteinase K, followed by genomic DNA extraction using the QIAamp DNA Mini Kit (Qiagen, Germany). Sequencing libraries were prepared using the NEBNext Ultra II FS DNA Library Prep Kit (MA, USA) and sequenced on Illumina iSeq100 or NextSeq2000 platforms with 150-bp paired-end reads (7) (Table 1).

**TABLE 1:** Details of the 14 bacteriophages sequenced in this study^*a*^^,^^*b*^

Sample ID	Accession no./ (SRA accession no.)	Date of collection (DD/MM/YYYY)	Sample location in Bangladesh (Latitude longitude)	Sample source	Size in bp (ORFs)	Genome coverage (G + C%)	Genus
CHRF_PH_0001	PQ336901 (SRR28112640)	21/08/2021	Dhaka (23.76 N 90.39 E)	Sewage	39,761 (65)	1,046× (51%)	*Kayfunavirus*
CHRF_PH_0002	PQ336902 (SRR28112639)	21/08/2021	Dhaka (23.75 N 90.40 E)	Lake	45,880 (87)	2,245× (46%)	*Macdonaldcampvirus*
CHRF_PH_0003	PQ336903 (SRR28112634)	21/08/2021	Dhaka (23.75 N 90.40 E)	Lake	37,939 (58)	273× (51%)	*Kayfunavirus*
CHRF_PH_0004	PQ336904 (SRR28112633)	22/08/2021	Dhaka (23.82 N 90.34 E)	Lake	38,164 (53)	1,013× (49%)	*Teseptimavirus*
CHRF_PH_0005	PQ336905 (SRR28112632)	22/08/2021	Dhaka (23.86 N 90.36 E)	River	38,546 (56)	881× (49%)	*Teseptimavirus*
CHRF_PH_0006	PQ336906 (SRR28112631)	23/08/2021	Dhaka (23.77 N 90.36 E)	Sewage	44,975 (84)	1,174× (46%)	*Macdonaldcampvirus*
CHRF_PH_0007	PQ336907 (SRR28112630)	23/08/2021	Dhaka (23.76 N 90.36 E)	Sewage	39,928 (63)	884× (51%)	*Kayfunavirus*
CHRF_PH_0009	PQ336908 (SRR28112629)	03/11/2021	Mirzapur (24.11 N 90.10 E)	Stagnant water	39,181 (61)	18,097× (51%)	*Kayfunavirus*
CHRF_PH_0011	PQ336909 (SRR28112628)	09/11/2021	Mirzapur (24.12 N 90.15 E)	Pond	38,099 (61)	11,083× (51%)	*Kayfunavirus*
CHRF_PH_0012	PQ336910 (SRR28112627)	08/01/2022	Chattogram (22.38 N 91.81 E)	Sewage	40,368 (65)	9,607× (51%)	*Kayfunavirus*
CHRF_PH_0013	PQ336911 (SRR28112638)	17/01/2022	Chattogram (22.35 N 91.85 E)	Sewage	39,889 (60)	6,989× (51%)	*Kayfunavirus*
CHRF_PH_0014	PQ336912 (SRR28112637)	06/03/2022	Chattogram (22.37 N 91.85 E)	Sewage	40,094 (61)	6,906× (51%)	*Kayfunavirus*
CHRF_PH_0015	PQ336913 (SRR28112636)	14/03/2022	Chattogram (22.37 N 91.80 E)	Sewage	39,834 (62)	8,555× (51%)	*Kayfunavirus*
CHRF_PH_0016	PQ336914 (SRR28112635)	14/03/2022	Chattogram (22.38 N 91.80 E)	Sewage	39,834 (62)	10,259× (51%)	*Kayfunavirus*

^*a*^ CHRF_PH_0001–CHRF_PH_0007 were sequenced in an Illumina iSeq100 and the CHRF_PH_0009, 0011–0016 were sequenced in a NextSeq2000 platform.

^*b*^ ORFs, open reading frames.

The quality of the sequencing reads was assessed using FastQC (version 0.11.5). Adapter sequences were trimmed using Trimmomatic (version 0.39) before assembled into single contig using Unicycler (version 0.4.9) (8). Assembled genomes were used for genus identification with Kraken2 (version 2.1.2) (9) and for protein function annotation with Pharokka (version 1.2.1) (10). The tail fiber and terminase genes were individually extracted for comparison of all Typhi phage genomes sequenced globally to date using a Biopython script (https://github.com/CHRF-Genomics/Phage_Analysis). These sequences were then aligned using Clustal Omega (11), and two separate phylogenetic trees were generated for each gene. All tools were run with default parameters.

All assembled phages resulted in near-complete genomes with an average coverage depth of over 1,000×, assessed using QUAST (version 5.2) (12). Of the 14 phages, 10 phages belong to the *Kayfunavirus* genus with 60–65 open reading frames (ORFs), 2 were categorized under *Macdonaldcampvirus* with 84–87 ORFs, and the remaining 2 under *Teseptimavirus*, containing 53–56 ORFs (Table 1).

**Fig 1:**
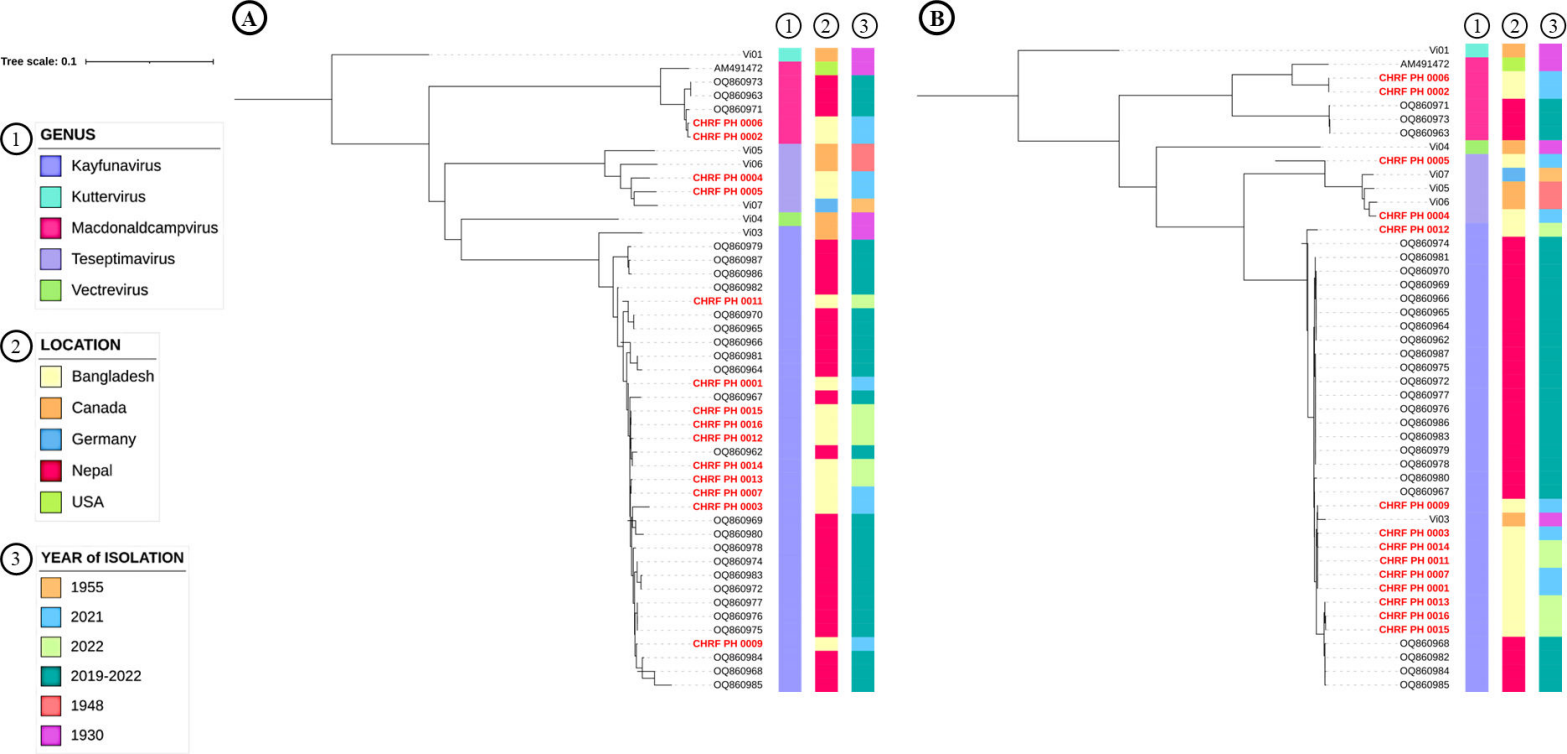
Phylogenetic tree of *Salmonella* Typhi bacteriophages. The 14 phages (highlighted in red) sequenced in this study were contextualized against 7 phages from the USA (13), Canada, and Germany (14) and 26 phages from Nepal (5). The genus, location, and year of isolation for the phage sequences are also shown. (**A**) Tail-fiber nucleotide sequences and (**B**) terminase nucleotide sequences of respective phages were extracted separately. These sequences were aligned, and separate phylogenetic trees were generated using Clustal Omega (online) (11). The resulting trees were visualized with iTOL (version 6.0) (15).

The phages sequenced in this study from Bangladesh were compared with 26 phages from Nepal (5) and 7 phages from the USA (13), Canada, and Germany (14). The majority of the phages from both Nepal and Bangladesh were identified as *Kayfunavirus*, covering three of the five genera of Typhi phages reported to date (5, 13, 14). Phylogenetic analyses based on the terminase and tail-fiber nucleotide sequences exhibited similar clustering patterns (Fig. 1), indicating that either gene can be used for comparative genomic analysis.

This study presents the near-complete genome sequences of *Salmonella* Typhi bacteriophages from Bangladesh, shedding light on the phage landscape in a region critically affected by typhoid fever.

## Data Availability

The raw data of all 14 Typhi phages in this study are available on NCBI under the BioProject PRJNA1081195. The phage genome sequences have been deposited in GenBank (accession numbers: PQ336901-14). Sequences of the 26 phages from Nepal are available under the BioProject PRJNA933946 (accession numbers: OQ860962-87). The sequences of the 7 phages from the USA, Canada, and Germany are available through NCBI (accession: AM491472) and Sanger FTP (ID: Vi01, Vi03-Vi07, from https://ftp.sanger.ac.uk/pub/project/pathogens/Phage/). The script used for extracting phage-specific protein sequences has been uploaded to https://github.com/CHRF-Genomics/Phage_Analysis.

## References

[1] Garrett DO, Longley AT, Aiemjoy K, Yousafzai MT, Hemlock C, Yu AT, Vaidya K, Tamrakar D, Saha S, Bogoch II, et al.. 2022. Incidence of typhoid and paratyphoid fever in Bangladesh, Nepal, and Pakistan: results of the Surveillance for Enteric Fever in Asia Project. Lancet Glob Health 10:e978–e988. doi:10.1016/S2214-109X(22)00119-X 35714648 PMC9210262

[2] Craigie J, Felix A. 1947. Typing of typhoid bacilli with Vi bacteriophage; suggestions for its standardisation. Lancet 1:823–827. doi:10.1016/s0140-6736(47)91719-4 20244895

[3] FELIX A. 1955. World survey of typhoid and paratyphoid-B phages types. Bull World Health Organ 13:109–170. https://iris.who.int/handle/10665/265572. 13260884 PMC2538040

[4] Freytag B, Plochmann E. 1955. Phage typing of typhoid strains. Z Hyg Infekt Krankh 142:188–196. doi:10.1007/BF02150088 13300415

[5] Shrestha S, Da Silva KE, Shakya J, Yu AT, Katuwal N, Shrestha R, Shakya M, Shahi SB, Naga SR, LeBoa C, Aiemjoy K, Bogoch II, Saha S, Tamrakar D, Andrews JR. 2024. Detection of Salmonella Typhi bacteriophages in surface waters as a scalable approach to environmental surveillance. PLoS Negl Trop Dis 18:e0011912. doi:10.1371/journal.pntd.0011912 38329937 PMC10852241

[6] Hooda Y, Islam S, Kabiraj R, Rahman H, Sarkar H, da Silva KE, Raju RS, Luby SP, Andrews JR, Saha SK, Saha S. 2024. Old tools, new applications: use of environmental bacteriophages for typhoid surveillance and evaluating vaccine impact. PLOS Negl Trop Dis 18:e0011822. doi:10.1371/journal.pntd.0011822 38358956 PMC10868810

[7] Islam S, Kabiraj R, Amin A, Hussain Pranto S, Dey S, Rajib Malaker A, Purna Keya D, Mohammad Tanmoy A, K Saha S, Hooda Y, Saha S. 2024. Protocol for genomic DNA extraction and sequencing library preparation from phage stock v1. Protocols.io. doi:10.17504/protocols.io.kqdg32477v25/v1

[8] Wick RR, Judd LM, Gorrie CL, Holt KE. 2017. Unicycler: resolving bacterial genome assemblies from short and long sequencing reads. PLOS Comput Biol 13:e1005595. doi:10.1371/journal.pcbi.1005595 28594827 PMC5481147

[9] Wood DE, Lu J, Langmead B. 2019. Improved metagenomic analysis with Kraken 2. Genome Biol 20:257. doi:10.1186/s13059-019-1891-0 31779668 PMC6883579

[10] Bouras G, Nepal R, Houtak G, Psaltis AJ, Wormald P-J, Vreugde S. 2023. Pharokka: a fast scalable bacteriophage annotation tool. Bioinformatics 39:btac776. doi:10.1093/bioinformatics/btac776 36453861 PMC9805569

[11] Sievers F, Higgins DG. 2018. Clustal Omega for making accurate alignments of many protein sequences. Protein Sci 27:135–145. doi:10.1002/pro.3290 28884485 PMC5734385

[12] Gurevich A, Saveliev V, Vyahhi N, Tesler G. 2013. QUAST: quality assessment tool for genome assemblies. Bioinformatics 29:1072–1075. doi:10.1093/bioinformatics/btt086 23422339 PMC3624806

[13] Pickard D, Thomson NR, Baker S, Wain J, Pardo M, Goulding D, Hamlin N, Choudhary J, Threfall J, Dougan G. 2008. Molecular characterization of the Salmonella enterica serovar Typhi Vi-typing bacteriophage E1. J Bacteriol 190:2580–2587. doi:10.1128/JB.01654-07 18192390 PMC2293211

[14] Pickard Derek, Toribio AL, Petty NK, van Tonder A, Yu L, Goulding D, Barrell B, Rance R, Harris D, Wetter M, Wain J, Choudhary J, Thomson N, Dougan G. 2010. A conserved acetyl esterase domain targets diverse bacteriophages to the Vi capsular receptor of Salmonella enterica serovar Typhi. J Bacteriol 192:5746–5754. doi:10.1128/JB.00659-10 20817773 PMC2953684

[15] Letunic I, Bork P. 2021. Interactive Tree Of Life (iTOL) v5: an online tool for phylogenetic tree display and annotation. Nucleic Acids Res 49:W293–W296. doi:10.1093/nar/gkab301 33885785 PMC8265157

